# Investigation into the protective ability of monovalent and bivalent A Malaysia 97 and A_22_ Iraq 64 vaccine strains against infection with an A/Asia/SEA-97 variant in pigs

**DOI:** 10.3389/fvets.2022.1027556

**Published:** 2022-10-28

**Authors:** Jacquelyn Horsington, Nagendrakumar Singanallur Balasubramanian, Charles K. Nfon, Hilary Bittner, Wilna Vosloo

**Affiliations:** ^1^Australian Centre for Disease Preparedness, Transboundary Disease Mitigation, Commonwealth Scientific and Industrial Research Organisation (CSIRO) Health and Biosecurity, Geelong, VIC, Australia; ^2^Canadian Food Inspection Agency, National Centre for Foreign Animal Disease, Winnipeg, MB, Canada

**Keywords:** foot-and-mouth disease, vaccines, A/Asia/SEA-97, protection, area under a curve, pigs

## Abstract

Over the last 15 years, FMDV serotype A viruses in South-East Asia (A/ASIA/SEA-97 lineage) have diverged into several clusters. Variants from Thailand in 2011-2013 have caused vaccine failures and returned poor r_1_-values (<0.30) to A_22_ Iraq 64 (A22) and A Malaysia 97 (A May) vaccine strains. We investigated the protective ability of monovalent and bivalent A Malaysia 97 and A22 Iraq 64 vaccine strains against infection with an A/Asia/SEA-97 variant in pigs. Pigs were challenged with a variant of A/Asia/SEA-97 lineage either 21- or 7- days post-vaccination (V21 or V7) using the heal-bulb challenge. Only one in five pigs were protected in the V21 monovalent vaccine groups. Less severe clinical signs were observed in the A22 IRQ group compared to the A MAY 97 group. In the V21 combination group, 4 out of 5 pigs were protected and viraemia was significantly reduced compared to the monovalent V21 groups. V7 vaccine groups were not protected. The neutralising antibody response was below the detection limit in all groups on the challenge day, showing a poor correlation with protection. There was no evidence that the pigs protected from systemic disease had protective antibody responses sooner than other pigs in the study, implying other immune mechanisms might play a role in protecting these animals. FMDV was detected in the nasal and oral swab samples between 1 and 6 dpc. Viral loads were lower in the nasal swab samples from the V21 combination group than the other groups, but there was no difference in the oral swab samples. Since all unvaccinated controls were euthanised by 6-day post-challenge for ethical reasons, the ‘area under the curve (AUC)' method was used to compare the viraemia and virus excretion in different groups. We recommend that for the A/Asia/SEA97 variants, a combination vaccine with A Malaysia 97 and A22 Iraq 64 vaccine strains would be ideal compared to monovalent vaccines.

## Introduction

Foot-and-mouth Disease (FMD) is an important transboundary animal disease of cloven-hoofed livestock and wild ungulates (1). It is highly infectious, has a high morbidity rate and causes significant loss in livestock production. Mortality due to the disease is seen predominantly in young animals. Most of the economic impact of FMD is because of restriction to trade with FMD-free countries, particularly where a trade barrier is imposed on livestock and their products (2). The disease is caused by the FMD virus (FMDV), a single-stranded, positive-sense RNA virus, which belongs to the genus *Aphthovirus* within the family *Picornaviridae*, and comprises the serotypes O, A, C, Asia1, and Southern African Territories (SAT) 1, 2 and 3 (3, 4).

FMDV is widely distributed and is currently maintained in three continental reservoirs: in Asia, Africa, and some parts of South America. The viruses in these continents are subdivided into seven major virus pools of infection that contain different serotypes and lineages (5). South-East Asia (SEA) is endemic to viruses belonging to Pool 1, comprising serotypes O and A, with a few historical occurrences of serotype Asia1. The viruses of SEA have evolved distinctly from the other regions in Asia with the emergence of O/SEA/CAM-94 lineage in 1994 and 1998 (6); A/ASIA/SEA-97 in 1997 (7) and O/SEA/Mya-98 in 1998 (8, 9) respectively. However, recently we have seen incursions of virus strains belonging to Pool 2 into the region, such as O/ME-SA/PanAsia and O/ME-SA/Ind-2001 (7).

Although there is heavy disease burden in SEA, the control of FMD has been hampered by many factors such as the geo-political situations, unrestricted animal movements, poorly resourced veterinary services, lack of funding for FMD control programs and producers' apathy (10). With increase in population and demand for animal protein, there is an increase in the number of small and marginal farmers who access transboundary markets through traders. This has resulted in unrestricted movements of animals within the region (11). Added to these problems is the non-availability of quality vaccines and poor vaccination rates. Different commercial vaccines incorporating different virus strains are sold in the region and there is insufficient data on vaccine matching in the region.

Thailand uses a locally manufactured vaccine with strains for serotype O and A that were previously collected in the country and that differs from other countries in the region. While the choice of serotype O vaccine strains depend on the different producers, there is some synergy in which serotype A vaccine strains are used in SEA. Both the vaccine strains, A/Sakolnakorn/97 and A/Malaysia/97, belong to the same genetic lineage and year of isolation, with the former isolated from the outbreaks in Thailand and the latter from Malaysia (25). These two strains continued to be a part of the FMD vaccines in the region for almost two decades. However, since 2012, serotype A viruses in the region, especially in Thailand, had diverged resulting in a distinct SEA-97 variant. These variants demonstrated a poor antigenic match with the serotype A vaccine strains (12). Some of these variants were antigenically so diverged that the routine antigen ELISA used in the region failed to detect them. Soon after the OIE-Regional Reference Laboratory for FMD (OIE-RRL), Pakchong in Thailand reported the emergence of this new SEA-97 variant, the National Laboratory for FMD, Thailand developed a new vaccine strain, A Lopburi 2012, from one of the isolates (13). This strain is now incorporated into the FMD vaccines manufactured in Thailand (13). Studies carried out at the OIE/FAO World Reference Laboratory (WRL) for FMD, the Pirbright Institute, United Kingdom, and at OIE-RRL, Pakchong indicated that these A/ASIA/SEA-97 variants from SEA had poor relative homology (r_1_) values in vaccine matching studies with the serotype A vaccines including A/Malaysia/97, but good matching with the new vaccine strain, A Lopburi 2012 (12–14).

Since viruses of this sub-lineage have spread to other countries such as Lao PDR, Vietnam, and Cambodia (12, 14), it was important to reassure stakeholders, including endemic countries in the region and free countries/regions holding vaccine banks, on the efficacy of the vaccine strains against these variant viruses. We report the results of the vaccine efficacy studies in pigs vaccinated with different serotype A vaccine strains and challenged by a field virus belonging to A/Asia/SEA-97 variant. Our aim was to establish if a monovalent vaccine or a combination vaccine would be effective in preventing clinical disease in pigs and if the vaccines will impart early protection soon after administration.

## Materials and methods

### Animals

Cross-bred landrace pigs 7–8 weeks of age and of mixed sex were obtained from a registered supplier in Canada. They were kept at the National Centre for Foreign Animal Diseases (NCFAD), Winnipeg, Canada, facility under quarantine for 2 weeks before the commencement of the experiment. This study was approved by the Australian Centre for Disease Preparedness (ACDP) Animal Ethics Committee (AEC 1774 and AEC 1801) and the Canadian Centre for Human and Animal Health Animal Care Committee (AUD# C-15-007) and performed in strict accordance with the recommendations of the Australian Code of Practise for the Care and Use of Animals for Scientific Purposes and the Canadian Council for Animal Care Guidelines.

### Vaccines and challenge virus

Monovalent A Malaysia 97 (A May) and A22 Iraq 64 (A22) double oil adjuvant vaccines with antigen payloads of at least 6 PD_50_/ml, and combination (Combo: A May and A22) double oil adjuvant vaccine with an antigen payload of at least 6 PD_50_ of each strain were prepared from the Australian FMD vaccine reserve by Merial Company Limited, United Kingdom (now Boehringer Ingelheim). The vaccines were imported to Canada and stored at NCFAD, Winnipeg, under controlled conditions.

The challenge virus, FMDV isolate A/TAI/15/2013, which belongs to the new A/Asia/SEA-97 variant, was obtained from the FAO/OIE World Reference Laboratory for FMD (WRL), Pirbright, United Kingdom. The virus was originally isolated from cattle on 21/10/2013 in the Lampang province of Thailand. Vaccine matching studies showed that the r_1_ values were 0.05 and 0.10 for A22 Iraq 64 and A Malaysia 97, respectively (14). It was passaged twice using BHK-21 cells at the WRL and imported into NCFAD. The isolate had a poor relative homology (r_1_) value of 0.05 and 0.01 against A22 Iraq 64 and A Malaysia 97 vaccine strains, respectively (14). To prepare for the pig challenge virus, the cell culture supernatant containing the isolate was passed once in two pigs by inoculation into the bulb of the heel (15, 16) of the left forelimb at two sites (0.1 ml/site), intravenously (1 ml) into the ear vein and intramuscularly (1 ml) on the mid-neck region as described (17, 18). Vesicular material was collected at 2 days post infection and a 10% w/v suspension prepared in phosphate buffered saline (PBS; pH 7.4 ± 2). The aliquots were stored at−80°C until use. One of the aliquots was titrated using LFBK-αVβ6 (αVβ6-expressing foetal porcine kidney) cells (19, 20).

### Study design

The vaccine efficacy experiment consisted of eight vaccine groups and unvaccinated controls (Supplementary Table 1) and was carried out in two phases. In Phase 1, the efficacy of the monovalent vaccines (A22 and A May) was studied in groups of five pigs and five control pigs and in Phase 2 the combination vaccine (A22 + A May) was tested in two groups of five pigs with five additional control pigs, resulting in ten unvaccinated control pigs (UVC). With each vaccine formulation, one group was vaccinated 21 days prior to challenge (V21) and one group was vaccinated 7 days prior to challenge (V7). Finally, a group of pigs was vaccinated either with A22 and A May vaccine (*n* = 2 each) or the Combo vaccine (*n* = 5), along with the V21 groups but were not challenged with virulent virus (VO). The vaccines were administered intramuscularly in the left side of the neck (2 ml/dose). Vaccination was staggered so that all pigs (except A May VO, A22 VO and Combo VO groups) were challenged on the same day.

All vaccinated pigs and the UVC group (except VO groups), were challenged by the heel bulb route using 0.2 ml of virus inoculum (equivalent to 10,000 TCID_50_) divided equally between two sites on one foot as previously described (17, 18). The animals were monitored for development of clinical signs consistent with infection by FMDV such as pyrexia (rectal temperature >40°C), lameness and development of vesicles on the surface of the tongue and snout, up to 6 days post-challenge (dpc). Lesion scores were calculated by scoring one for each site where lesions formed, except the inoculation site (1 per foot and 1 for any oral/snout lesions) resulting in a maximum score of 4. Whole blood in K2-EDTA vials was collected from all pigs at the time of vaccination,−4 dpc and daily between 0 and 14 dpc, at which point the experiment was terminated for RT-qPCR. Clotted blood for serology was collected from pigs in VO groups on all days synchronous with the challenge groups i.e.,−21 dpc,−7 dpc, 0 dpc, 5 dpc, 7 dpc, 10 dpc and 14 dpc, corresponding to 0-, 14-, 21-, 26-, 28-, 31- and 35-day post-vaccination (dpv). The serum was inactivated at 56°C for 30 min and stored in aliquots under −70°C until use. Small, sterilised cotton buds were used to collect nasal and saliva secretions daily between 0 and 14 dpc for virus isolation and RT-qPCR. Swabs were placed in tubes containing 500 μl of PBS for RT-qPCR or 500 μl Dulbecco's modified Eagle's media (DMEM) containing 5% foetal bovine serum and antibiotics (Gibco, Cat. No. 15240062) for virus isolation. All samples were stored at −70°C until processing.

Pigs were sedated using isoflurane gas anaesthesia, during heel bulb inoculation and collection of samples. If deemed necessary, Flunixin Meglumine (1.1–2.2 mg/kg) and Buprenorphine (0.005–0.01 mg/kg) was administered every 12–24 h to manage pain. Pigs were humanely euthanised when they reached the ethical end points or end of the experimental period; pigs were sedated first (0.8 mL xylazine at 20 mg/ml and 4.5 mL ketamine at 100 mg/ml) followed by intravenous barbiturate injection (sodium pentobarbital at 100 mg/kg).

### Virus isolation

Serum, nasal swab, and saliva (oral swab) samples were tested for the presence of live virus using LFBK-αVβ6 cells [LFBK cells; (19, 20)]. Monolayers of LFBK cells grown in 96-well cell culture trays were inoculated with 100 μl sample and incubated for 30 mins at 37°C. The cells were washed with PBS and overlayed with DMEM containing 5% foetal bovine serum and antibiotics (Gibco, Cat. No. 15240062) and examined for cytopathic effect (CPE) after 24, 48 and 72 h incubation at 37°C with 5% CO_2_. If no CPE was observed, cells and supernatant were collected, freeze-thawed and inoculated onto fresh LFBK monolayers. The presence or absence of FMDV was confirmed using an FMDV antigen enzyme-linked immunosorbent assay (ELISA) as described by Hamblin et al. (21).

### Detection of FMDV RNA by RT-qPCR

The FMDV RNA levels in serum, nasal and oral swabs were quantified by a TaqMan RT-qPCR assay as described previously (22). Viral RNA was extracted from 50 μl of sample with the MagMAX™-96 Viral RNA Isolation Kit (Life Technologies) using the MagMAX™ Express-96 Magnetic Particle Processor (Life Technologies). One-step RT-qPCR was performed using the AgPath ID One-Step RT-PCR reagents (Life Technologies) on the Applied Biosystems 7500 Real-Time PCR Instrument. All samples were tested in duplicate and samples with poor Ct value correlation in the duplicate reactions were repeated. Samples with a Ct <40 (equivalent to 1 × 10^3.5^ copies RNA/ml blood or 1 × 10^3.2^ copies RNA/swab) were considered positive (23).

### Determination of neutralising antibody titre

Virus neutralisation test (VNT) in LFBK cells was performed on heat inactivated (56°C, 30 min) serum samples using either A/MAY/97 or A22/IRQ/64 virus (provided by NCFAD) and the LFBK-αVβ6 cell adapted A/TAI/15/2013 virus (24). Titres >1.2 log10 (1:16) were considered positive (25).

### Detection of antibodies to structural proteins by ELISA

The presence of antibodies against structural proteins (SP) of serotype A was assayed using a serotype A-specific solid-phase competition ELISA (SPCE) using reagents homologous to A22/IRQ/64 following a protocol described by Mackay et al. (26) with some modifications with respect to the antigens and control sera. We did not have a system with homologous reagents for A Malaysia 97 and so not performed.

### Statistical analysis

Data on clinical scores, virus RNA levels and VNT antibody titres were used for statistical analysis using R version 4.0.2 (27). Clinical protection based on count data was compared using the two-sided Fischer exact test. Group means and standard deviations were calculated and expressed as Mean ± SD. Mean survival time and probability of protection were estimated using Kaplan-Meier survival analysis [(28), “*survival*” and “*survminer*” libraries in R]. Longitudinal data for continuous outcomes in multiple vaccine groups were compared using a linear mixed effects model (“*lme”* library in R). All plots were drawn using the library “ggplot2” in R. ANOVA was used to test the statistical differences between groups with Holm's *post-test* if a statistical difference was found. Longitudinal data (virus isolation, RT-PCR results and NSP response) were analysed using animal number as random variable and dpc, group and vaccination (yes or no) as possible explanatory variables. Using forward selection, the best model with the lowest AIC (Akaike's Information Criterion) was chosen. Pig number was added as a random variable while dpc (as a factor) and vaccine group and the interactions were considered as explanatory variables. In all models, explanatory variables were selected based on the lowest AIC using forward selection. Area under the curve (AUC) was used to compare estimated virus loads in serum, nasal and oral swabs, and the duration of viraemia in a single parameter (29, 30). A new variable, AUC units, was constructed to measure the FMDV load in pigs from day of challenge to end of experiment or removal of pig in terms of duration and quantity of excretion (log10 copy numbers/ml). The median and mean AUC units for each animal were calculated following the trapezoidal rule using “*rgeos*” and library in R. Group-wise comparison of median and mean AUC units were performed using one way ANOVA with *post hoc* Bonferroni's test (31) using “*car*” library in R.

## Results

### Clinical signs

**Figure 1:**
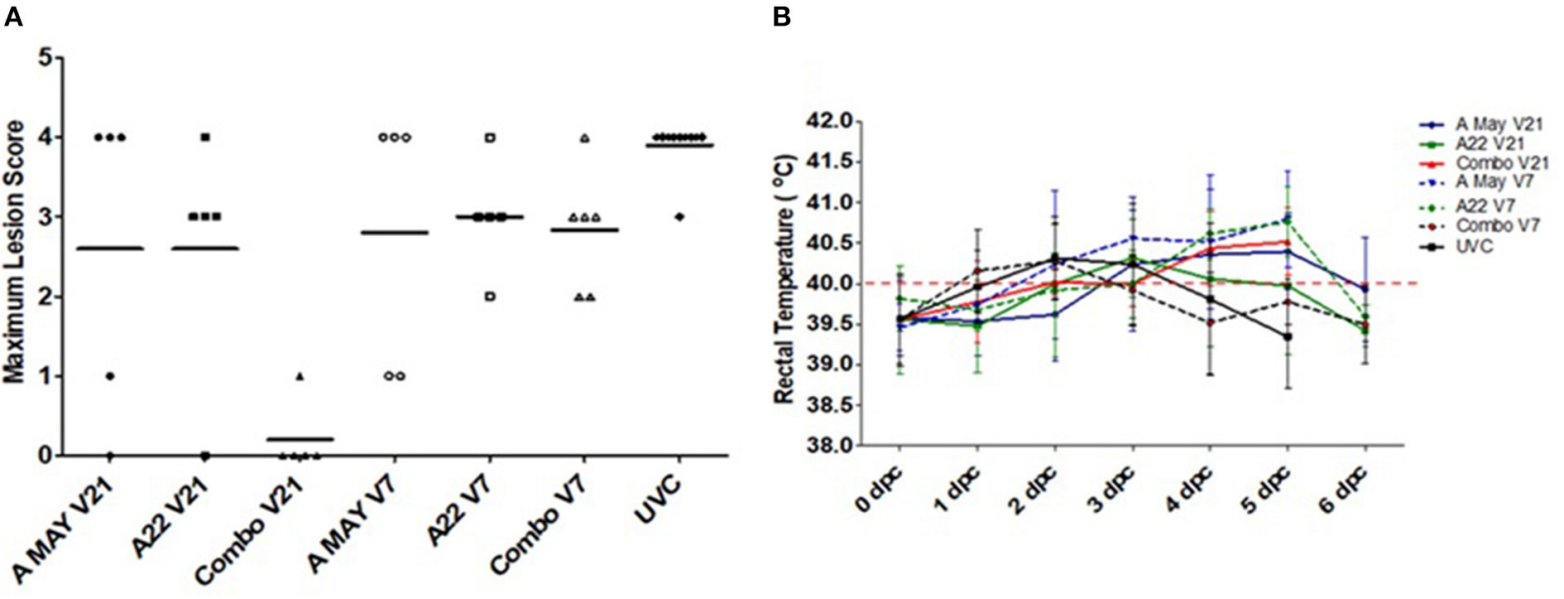
Lesion scores and rectal temperatures. **(A)** Maximum lesion scores in pigs challenged with A/Asia/SEA-97 variant virus post-vaccination. Horizontal lines indicate the group mean scores. **(B)** Average rectal temperatures in pigs vaccinated and challenged during the experiment. Temperatures >40°C were considered Horpyrexia (red dotted line).

All vaccinated and unvaccinated pigs, except those in the VO group, were challenged with A/TAI/15/2013 and monitored for up to 14 dpc. All unvaccinated control pigs developed clinical signs and since they reached the ethical endpoint, they were euthanized between 3 and 5 dpc. Systemic disease (vesicular lesions on the non-inoculated feet, snout and/or tongue) was observed in all V7 pigs, regardless of vaccine used. Several animals in these groups were euthanized between 4 and 6 dpc after reaching humane endpoint. The mean maximum lesion score was lower in all vaccinated groups compared to the control group (Figure 1A), however, when the different vaccine groups were compared, significant protection was only observed in the Combo V21 group (4/5 pigs protected: Fisher's Exact *p* = 0.01794). In the A May V21 and A22 V21 groups, 1/5 pigs were protected from systemic disease (Figure 1B). The individual daily lesion scores and time of euthanasia are presented in Table 1). Elevated rectal temperatures lasting 2–4 days were observed in the pigs in all groups between 1 and 6 dpc (Figure 1B).

**Table 1:** The individual lesion scores for the first 7 dpc and the time of euthanasia as well as the protection outcome.

**Vaccine**	**Day of vaccination**	**Group**	**Pig ID**	**0 dpc**	**1 dpc**	**2 dpc**	**3 dpc**	**4 dpc**	**5 dpc**	**6 dpc**	**7 dpc**	**Status**
A Malaysia 97	−21 dpc^†^	A May V21	001	0	0	3	3	4	E	E	E	Not protected
			002	0	0	2	3	4	2	E	E	Not protected
			003	0	0	0	0	0	0	0	0	Protected
			004	0	0	4	4	4	4	4	E	Not protected
			005	0	0	0	0	0	1	1	E	Not protected
A22 Iraq 64	−21 dpc	A22 V21	006	0	0	0	2	3	2	3	E	Not protected
			007	0	0	0	0	0	0	0	0	Protected
			008	0	0	0	1	3	2	2	2	Not protected
			009	0	0	1	2	4	4	E	E	Not protected
			010	0	0	1	1	2	3	2	E	Not protected
A Malaysia 97 + A22 Iraq 64	−21 dpc	Combo V21	030	0	0	0	0	1	1	1	1	Not protected
			031	0	0	0	0	0	0	0	0	Protected
			032	0	0	0	0	0	0	0	0	Protected
			033	0	0	0	0	0	0	0	0	Protected
			034	0	0	0	0	0	0	0	0	Protected
A Malaysia 97	−7 dpc	A May V7	011	0	0	3	4	4	4	E	E	Not protected
			012	0	0	0	1	1	1	E	E	Not protected
			013	0	0	3	4	4	4	E	E	Not protected
			014	0	0	0	0	1	1	E	E	Not protected
			015	0	0	3	4	4	E	E	E	Not protected
A22 Iraq 64	−7 dpc	A22 V7	016	0	0	0	3	3	1	E	E	Not protected
			017	0	0	0	1	2	2	E	E	Not protected
			018	0	0	0	0	1	2	2	2	Not protected
			019	0	0	0	1	1	1	1	E	Not protected
			020	0	0	3	3	3	3	E	E	Not protected
A Malaysia 97 + A22 Iraq 64	−7 dpc	Combo V7	035	0	0	0	0	1	2	E	E	Not protected
			036	0	0	0	0	2	3	E	E	Not protected
			037	0	0	0	0	2	3	E	E	Not protected
			038	0	0	0	3	4	4	E	E	Not protected
			039	0	0	0	1	2	3	E	E	Not protected
Unvaccinated controls		UV	021	0	0	3	4	4	E	E	E	Not protected
			022	0	0	4	4	4	E	E	E	Not protected
			023	0	0	3	4	4	E	E	E	Not protected
			024	0	0	3	3	4	E	E	E	Not protected
			025	0	0	3	4	4	E	E	E	Not protected
			040	0	0	4	4	E	E	E	E	Not protected
			041	0	0	2	3	3	3	E	E	Not protected
			042	0	0	3	4	4	4	E	E	Not protected
			043	0	0	4	4	4	E	E	E	Not protected
			044	0	0	3	4	3	E	E	E	Not protected

^†^dpc, days post-challenge; Score of 1 for each site where lesions form besides inoculation site (maximum of 4). E, euthanized (animals where E is not indicated were euthanized at the end of the study, 14 dpc).

**Figure 2:**
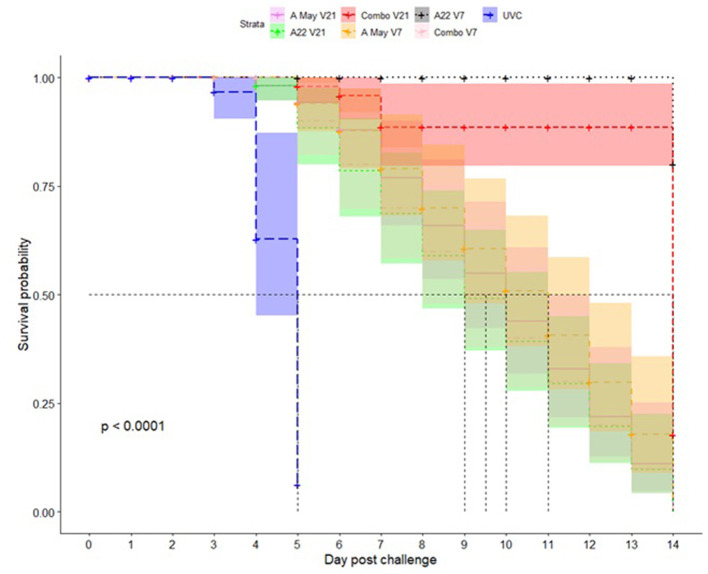
Kaplan-Meier survival plots for protection against A/TAI/15/2013 virus in pigs vaccinated with A May 97 and A22 Iraq 64 vaccines in monovalent or combination formulations. The x-axis represents time in days, and the y-axis shows the probability of surviving or the proportion of pigs surviving the virus challenge post-vaccination in different vaccine groups. The lines represent survival curves of the seven vaccine groups (Vaccine = 1: A May V21; Vaccine = 2: A22 V21; Vaccine = 3: Combo V21; Vaccine = 4: A May V7; Vaccine = 5: A22 V7; Vaccine = 6: Combo V7; Vaccine = 7: UVC). A vertical drop in the curves indicates an event (pigs showing clinical signs of FMD). The vertical tick mark on the curves means that a pig was censored at this time.

Kaplan-Meier survival analysis was performed on the day pigs showed lesions post-challenge in each group; the mean probability of protection (0-1) and median time to appearance of lesions was estimated (as dpc). In the UVC group the median time until lesions appeared was 5 dpc (4–5 dpc) and the mean probability of protection was 0.06 (0.01-0.42; mean and 95% CI) by 5 dpc. The pigs in the Combo V21 group performed the best with the median protection time until lesions develop indeterminable (∞ dpc) and the mean probability of protection from clinical disease 0.80 (0.52–1.00; mean and 95% CI) by 14 dpc. For the other vaccine groups, the median time until lesions appeared was between 9 (A May V7) and 14 days (A22 V21). For the A May V21 group the median time was 10 days compared to 11 days for the A22 V7 group. The probability of protection progressively decreased from 5 dpc to 14 dpc (Figure 2, Supplementary Tables 2A,B).

### Detection of neutralising antibodies using VNT in the challenged groups

Complete results of the homologous and heterologous (challenge virus) neutralising antibody titres in different vaccine groups are in Supplementary Tables 3A–C. At the time of challenge (21 or 7 dpc), none of the vaccinated pigs had measurable homologous or heterologous neutralising antibodies to the vaccine or challenge strain. As a result of the pigs that reached endpoint and had to be euthanised, only limited data were available to investigate the anamnestic response up to 14 dpc.

### Detection of antibodies to structural proteins using an FMD A22 CELISA in the challenged groups by SPCE

**Figure 3:**
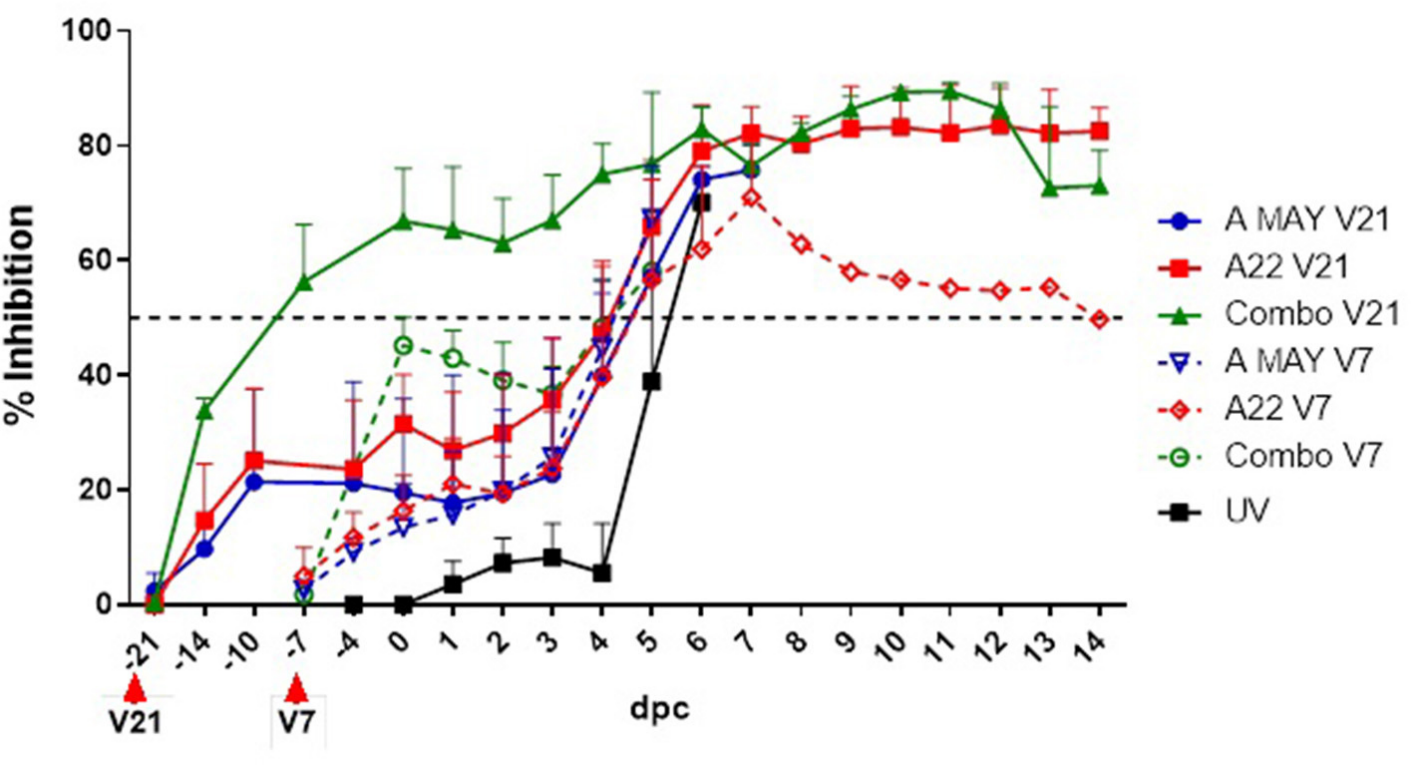
Antibody responses to FMDV structural protein based on a solid-phase competition ELISA. Per cent Inhibition values are shown in the y-axis and day post-challenge (dpc) in the x-axis. The error bars represent the standard deviation of mean PI values. The horizontal dashed line indicates the cut-off level for a sample to have a positive response to vaccination. V21 and V7 indicate the day the vaccines were administered prior to the challenge.

Antibodies to FMDV A structural proteins were detected by SPCE using A22 as antigen (Supplementary Table 4, Figure 3). A stronger antibody response was observed in the Combo V21 pigs compared to the A May V21 or A22 V21 pigs post-vaccination. All Combo V21 pigs and two of the A May V21 pigs were seropositive at the time of challenge. None of the V7 pigs had seroconverted by the day of challenge. An anamnestic response was observed in the vaccinated pigs from 3 dpc, when compared to the UV controls.

### Antibody responses in unchallenged vaccinated pigs (VO groups) using VNT and SPCE

At the same time as the groups that were vaccinated 21 days prior to challenge, two pigs each were vaccinated with A22 and A May 97 vaccines (A22 VO and A May VO respectively). An additional 5 pigs were vaccinated with the Combo vaccine (Combo VO). There animals were not challenged but their antibody titres were measured at the same days as the V21 challenge groups.

The neutralising antibody titres to A May 97, A22 and A TAI/15/2013 are presented in Supplementary Tables 3A–C. The A May VO group did not have homologous or heterologous antibody titres throughout the course of the experiment. One of the A22 VO group animals had low detectable homologous antibody titres by 7 dpc (28 dpv) and both had variable levels of heterologous antibody titres to A/TAI/15/2013 by 31 dpv and 35 dpv.

In the Combo VO group, the homologous and heterologous responses were generally low with a small number of pigs having measurable antibodies by 7 dpc (28 dpv). One pig (#046) did not sero-convert up to 35dpv.

With the SPCE assay that used A22 specific reagents, the animals in the VO groups had detectable antibodies as early as 25 dpv (−4 dpc) in most of the animals (6/9 pigs) and by 35 dpv (14 dpc) eight out of nine pigs had seroconverted (Supplementary Table 4). Using this assay, antibodies were detected in pig #046.

### Viraemia

Viraemia was detected by both real-time RT-qPCR and virus isolation (Table 2). Infectious virus was detected in only four of the vaccinated pigs up to 3 dpc, except in groups Combo V21, A22 V7 and Combo V7.

**Table 2:** Viral RNA concentration measured as log_10_ copy numbers/ml by a reverse transcription and quantitative PCR (RT-qPCR) in sera from animals challenged with A/TAI/15/2013 virus.

**Group**	**Pig ID**	**0 dpc^†^**	**1 dpc**	**2 dpc**	**3 dpc**	**4 dpc**	**5 dpc**
A May V21	001	-	4.91*	7.02	6.59	3.92	E
	002	-	4.33	3.55	5.43	4.05	-
	003	-	-	-	3.76	-	-
	004	-	5.65	7.21	4.68	-	-
	005	-	-	-	-	-	-
A22 V21	006	-	-	3.76	-	-	-
	007	-	3.78	-	-	-	-
	008	-	-	-	-	-	-
	009	-	-	-	5.69	4.93	-
	010	-	-	-	3.76	-	-
Combo V21	030	-	-	-	-	4.51	-
	031	-	-	-	-	-	-
	032	-	-	-	-	-	-
	033	-	-	-	-	-	-
	034	-	-	-	-	-	-
A May V7	011	-	4.25	6.04	5.55	-	-
	012	-	-	3.82	3.76	-	-
	013	-	-	5.52	4.49	-	-
	014	-	-			-	-
	015	-	-	6.17	5.64	-	E
A22 V7	016	-	3.76	-	-	-	-
	017	-	-	-	-	-	-
	018	-	-	-	4.09	-	-
	019	-	-	-	-	3.81	-
	020	-	4.66	5.50	-	-	-
Combo V7	035	-	-	-	-	-	-
	036	-	-	-	4.86	4.60	-
	037	-	-	4.91	4.65	-	-
	038	-	-	-	4.39	-	-
	039	-	5.09	-	5.41	4.83	-
UV	021	-	4.03	7.74	6.17	3.97	E
	022	-	6.28	8.22	6.76	5.41	E
	023	-	5.76	8.71	6.53	4.06	E
	024	-	5.07	8.26	6.68	4.22	E
	025	-	5.11	8.93	6.13	-	E
	040	-	6.75	8.16	7.18	E	E
	041	-	7.04	9.22	5.87	5.51	E
	042	-	7.63	9.93	6.99	6.16	-
	043	-	8.04	9.53	4.48	4.89	-
	044	-	6.43	8.44	4.96	4.55	E

^†^dpc, day post-challenge; *Log_10_ FMDV RNA copies/ml serum; Red square = VI positive; E = animal had been euthanized.

Samples positive by virus isolation on LFBK_α5β6_ cells are highlighted with grey colour. - indicates samples where the viral RNA was below the detection limit of the RT-qPCR.

**Figure 4:**
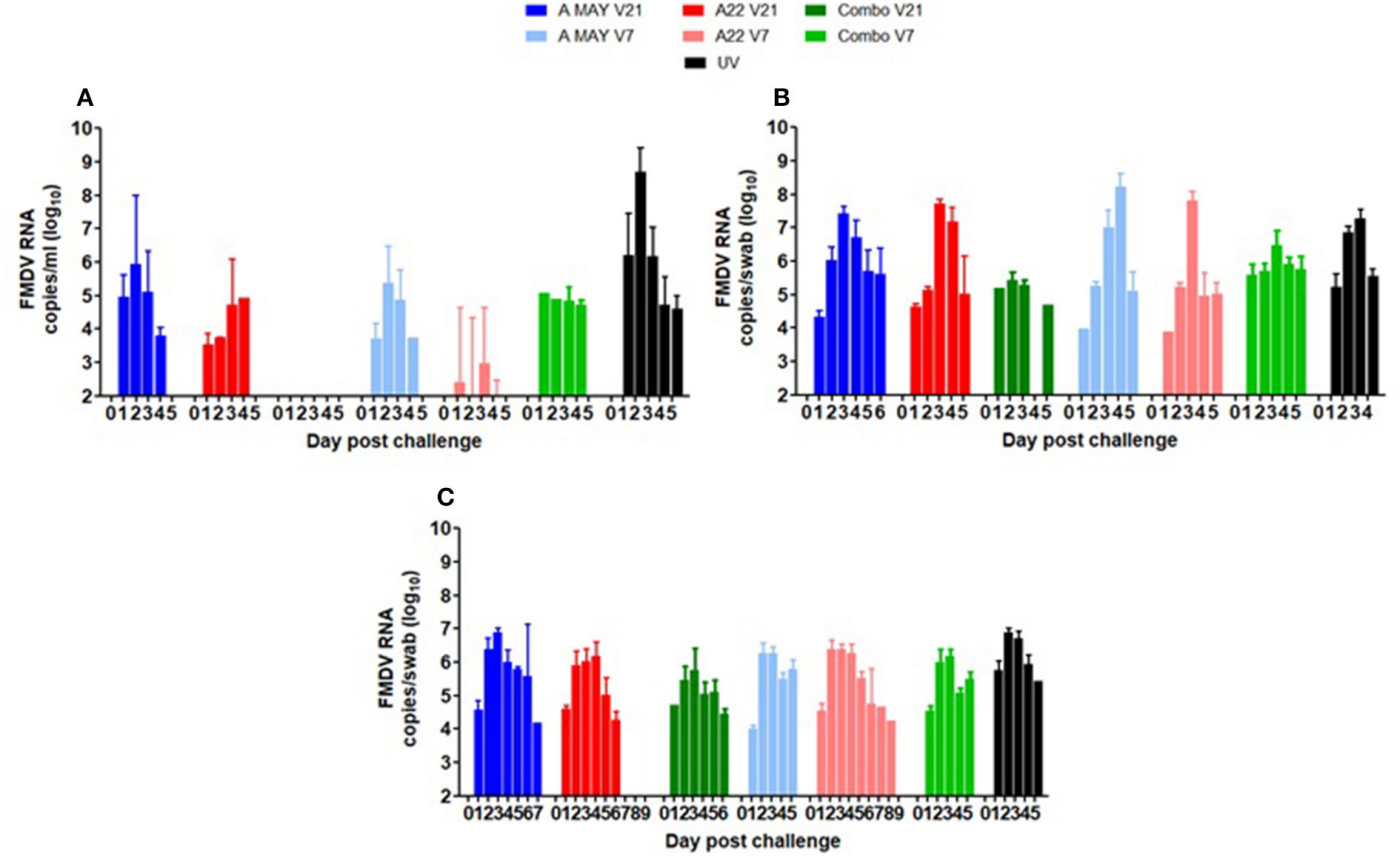
Mean viral loads as **(A)** Log_10_ FMDV RNA copies/ml in serum indicative of viraemia and Log_10_ FMDV RNA copies/swab indicative of virus shedding in **(B)** oral swabs and **(C)** nasal swabs. The error bars show the standard deviation of the mean. Since many pigs were euthanised due to the appearance of lesions and attaining humane endpoints, the numbers in the day post-challenge differ with different groups.

FMDV RNA was detected in the serum of most animals on at least 1 day between 1 and 4 dpc, except for the Combo V21 group, where only one pig was positive on 1 day, 4 dpc (Table 2; Figure 4A). The highest mean viraemia was in the UV pigs with FMDV RNA detected in all animals during 1–4 dpc with most pigs in this group removed from the study on 5 dpc. No viral RNA was detected in the blood of any vaccinated pigs after 4 dpc (not shown in Figure 4A). With 21 days between vaccination and challenge, the combination vaccine was most effective at reducing viraemia and the A May vaccine appeared less effective at reducing viraemia compared to the A22 vaccine. There was statistically insignificant difference in viral RNA between the groups challenged−7 dpv (*P* < 0.05). Peak viraemia was observed as early as 2 dpc (*p* = 0.005) and 3 dpc (*p* = 0.045) in A May V7 vaccine group when compared to the other vaccinated groups. All vaccinated animals showed a decrease in detectable viraemia compared to the unvaccinated animals (*P* < 0.001).

### Excretion of virus in nasal and oral swabs

FMDV RNA was detected in nasal and oral swabs from all pigs between 1 and 7 dpc. Viral RNA levels in oral swabs were similar in all groups (UVC and vaccinated and challenged; Figure 4B). Peak excretion in nasal secretion was observed at 3 and 4 dpc (*p* = 0.004) in all groups (Figure 4C). Compared to the other vaccine groups reduced virus excretion in nasal swabs was observed in the Combo 21 group (*p* < 0.001; Figure 4C). As many of the pigs from the vaccinated and UVC groups animals were removed at 4 or 5 dpc, comparison of duration of excretion between groups was not possible.

### Statistical comparison of median and mean AUC values for viral RNA in serum, oral and nasal secretions

**Figure 5:**
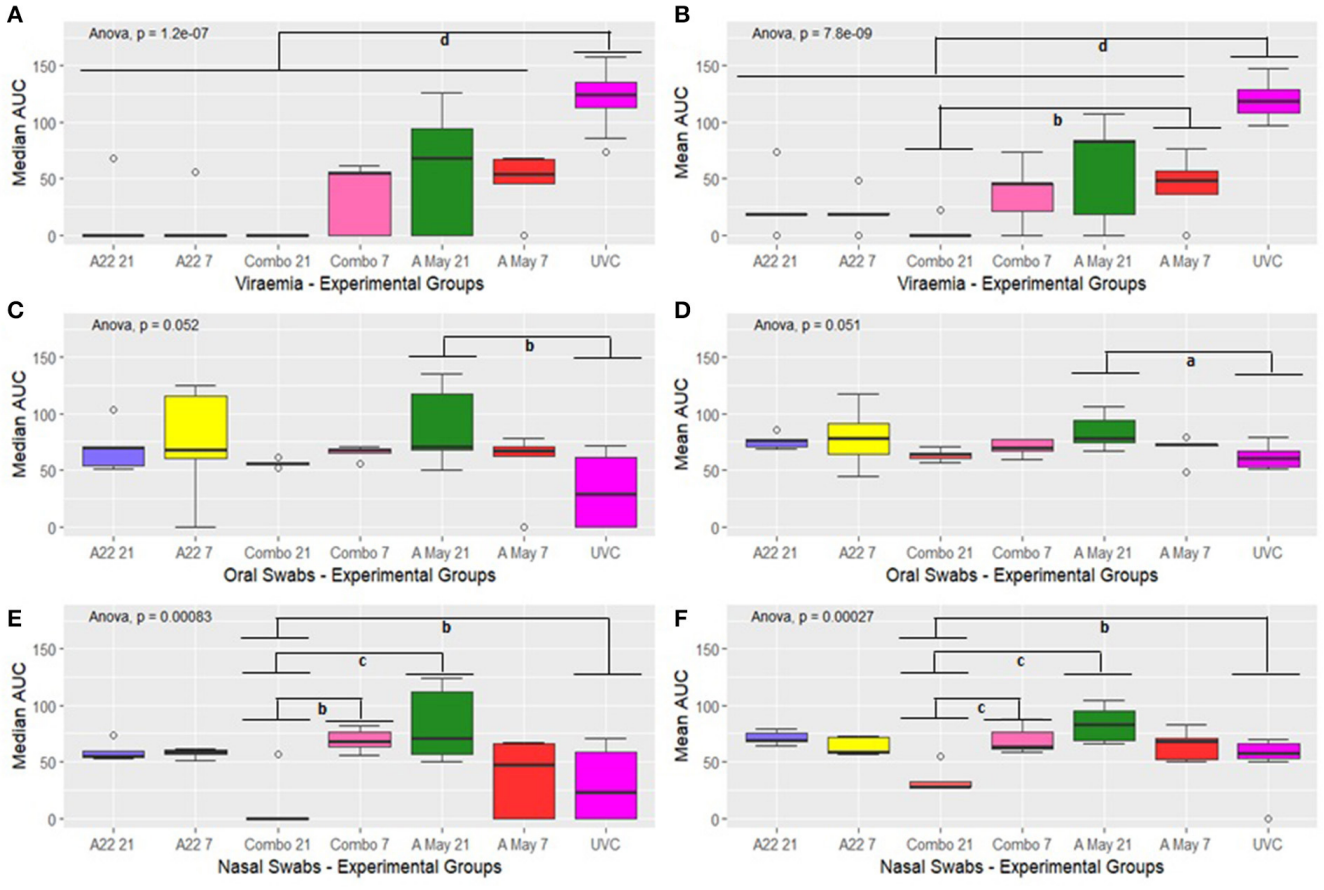
Box plot showing median and mean AUC of virus load in the blood **(A,B)**, oral fluids **(C,D)** and nasal secretions **(E,F)**. The boxplots show the interquartile range (median represented as the thick horizontal line within the box, and the first and third quartile of the data) and the minimum and maximum values for each group connected to the boxes with the vertical line. Groups with subscripts differ significantly. a = *p* < 0.1; b = *p* < 0.05; c = *p* < 0.01 and d = *p* < 0.001.

Due to ethical reasons, several pigs from each group were euthanised before the end of the trial at 14 dpc and the Area Under Curve was used to compare the viral RNA loads in serum, nasal, and oral swabs post-challenge. The median and mean AUC values for levels of viral RNA in blood, nasal and oral secretions were estimated for individual pigs in each group (Supplementary Tables 5A–C) and between group comparisons were performed using one-way ANOVA (including all groups) or two-way ANOVA (excluding the UVC group). The results of ANOVA on the median and mean AUC for viraemia, virus excretion in nasal and oral secretions are presented in Figures 5A–F and Supplementary Tables 6A,B.

A significant difference in the median and mean AUC value for viral RNA in sera was observed in the different groups (one-way ANOVA *p* = 1.2e-07 and *p* = 7.8e-09 respectively, Figures 5A,B) and the difference was attributable to the differences between the vaccine and the control groups (*p* < 0.001). A significant difference in mean viral RNA in the blood was also present between the Combo V21 and A May V7 groups (Figure 5B, *p* < 0.05).

The overall differences in median and mean values for viral RNA in the oral swabs were significant between the different groups (ANOVA *p* = 0.052 and *p* = 0.051 respectively; Figures 5C,D). *Post hoc* tests with Bonferroni correction (Supplementary Table 5A) showed that the median AUC values differed significantly between the A May V7 and UVC group only (*p* < 0.05) and the mean AUC values were also significantly different (*p* < 0.1).

The median and mean AUC values for the nasal swabs differed between various experimental groups (*p* = 0.00083 and *p* = 0.00027 respectively; Figures 5E,F). The values for the Combo V21 group differed significantly from the Combo V7 (*p* < 0.05 and *p* < 0.01 respectively; Supplementary Table 5A), A May V21 (*p* < 0.01 and *p* < 0.01 respectively) and the UVC group (*p* < 0.05 and *p* < 0.05 respectively; Supplementary Table 5A).

Since we lost all the unvaccinated control pigs by 6 dpc, we compared the median and mean AUC values in vaccinated groups excluding the unvaccinated groups to identify differences in viraemia and viral excretion using a two-way ANOVA (vaccine vs challenge day i.e., V21 or V7). The results are presented in Supplementary Table 5B. The AUC values for viraemia in vaccine groups differed significantly (*p* = 0.0728 and *p* = 0.090 resp.) and the difference was prominent between A May 97 vs. Combo vaccine groups (V21; adjusted *p* = 0.098 and 0.062 resp.). There was no significant difference in the AUC values for virus excretion in oral swabs between the vaccine groups (*p* < 0.100). However, significant differences were noticed in the median AUC values for virus excreted in the nasal secretions (*p* < 0.0001) and the difference was prominent between the Combo groups (V21 vs V7; adjusted *p* = 0.006) and between A May 91 and Combo groups (V21; adjusted *p* = 0.0006). The mean AUC values were also significantly different (*p* < 0.0001) and could be attributed to Combo V21 vs A22 V21 (adjusted *p* = 0.0006), Combo V7 vs A22 V7 (adjusted *p* = 0.00708), Combo V21 vs V7 (adjusted *p* = 0.001), A May 97 and Combo (V21; adjusted *p* < 0.0001) and A May 97 V7 vs. Combo V21 (adjusted *p* = 0.0045).

## Discussion

The emergence of novel variants of FMDV in South and South-East Asia continues to pose a threat to Australia's biosecurity and livestock industries. The continued evolution of O/ME-SA/Ind2001 lineage and spread to FMD-free countries like Indonesia and the appearance of new sublineages of A/Asia/SEA-97 in the region are of grave concern. Unlike serotype O there are limited vaccine strain options for serotype A, due to significant antigenic diversity and lack of cross protection. Therefore, it is important to continuously evaluate the existing vaccine strains against variants emerging in the region. The emergence of a new Thai variant of A/Asia/SEA-97 lineage is a serious threat and there is a paucity of information if the internationally recognised vaccine strains, A Malaysia 97 and A22 Iraq 64, would offer protection in animals infected with this sub-lineage of viruses. In this study, we compared the outcomes of virus challenge at 7 and 21 dpv in pigs vaccinated with the above-mentioned vaccine strains as monovalent vaccines or as a combined vaccine.

The challenge virus, A/TAI/15/2013, was highly virulent in pigs and for ethical reasons many pigs had to be euthanised before the end of the study. This resulted in fewer animals for all observable time points. It was challenging to obtain a meaningful outcome when comparing the viraemia and virus shedding in nasal and oral secretions between the different groups using traditional statistical methods (ANOVA). A method of using AUC of the viral load integrated over time was used in this study to compare the response in pigs vaccinated and challenged with FMDV with unvaccinated and challenged pigs. The AUC approach is a universal means of assessing the interrelationship among initial viral load, rate of increase of viral load and peak viral load (32). By combining the absolute viral load and the duration of viraemia into a single parameter, the AUC concept provides a means of combining the determinants of viraemia (29). This approach was shown to be a valuable method to access PCV2 infections in pigs and their effect on the average daily weight gain and viral load. Such a method was used to compare virus loads in patients infected with Respiratory Syncytial Virus in the past (33). Comparisons were also made between the AUC during viraemia and the absolute virus load for cytomegalovirus infections in human patients undergoing kidney transplants (29). Since this method considers the duration of viraemia/shedding and the load of the virus we can map the success of the vaccine-induced protection in pigs when pigs are removed from experiments due to ethical reasons or on reaching the humane endpoint. The assumption is that not only the virus load but also the duration of shedding is important when the vaccine quality is assessed.

Vaccination with high potency A22 Iraq or A May 97 protected 20% of pigs from clinical FMD following challenge with FMDV/A/TAI/15/2013 when administered 21 days prior to challenge. Overall, clinical signs were less severe in animals that received the A22 Iraq vaccine compared to those that received the A May 97 vaccine in both V7 and V21 groups. The A22 vaccinated pigs also had a reduced viraemia when compared to the A May 97 vaccinated pigs. In contrast, using the combination A22/A May vaccine, 80% (4 of 5) of the V21 pigs were protected from disease. Neither vaccine protected pigs when administered just 7 days prior to the challenge. All V7 pigs developed a systemic disease and were euthanized between 3 and 5 dpc. Viraemia was notably reduced in the Combo V21 pigs (only one animal positive), however, in the Combo V7 pigs, results were comparable to those seen in the A May V7 and A22 V7 pigs.

None of the vaccines induced a protective neutralising antibody response by the time of challenge, 7 or 21 dpv, showing poor correlation between neutralising antibody levels and protection. There was also poor correlation between neutralising titres and SP ELISA results. In ELISA, all Combo V21 pigs and two A May V21 pigs had seroconverted by the day of challenge; however, these results were also not entirely concordant with clinical protection. As most vaccinated pigs had seroconverted by 4 dpc, and only 1/10 UV pigs had seroconverted at this time, there is some indication of an anamnestic response, however with many pigs culled at 4 or 5 dpc, this result is not conclusive.

Virus was detected in the nasal and oral swab samples from all pigs between 1 and 6 dpc. Viral loads were lower in the nasal swab samples from the Combo V21 pigs compared to the other groups, but there was no difference in the oral swab samples.

These results suggest the combination A22 Iraq/A May 97 vaccine is more effective at providing protection from the A/TAI/19/2013 strain than the individual strain vaccines, with 21 days between vaccination and challenge. Kaplan-Meier survival analysis showed that a combination vaccine with A22 and A May 97 vaccine strains will be suitable for use against A/Asia/SEA-97 variants with a high probability of protection followed by A22 monovalent vaccine.

There was no evidence that the pigs protected from the systemic disease had protective antibody responses sooner than other pigs in the study, implying other immune mechanisms might play a role in the protection of pigs. All Combo V21 pigs and two of the A May V21 pigs were seropositive at the time of challenge. We did not have a homologous ELISA system with A May 97 reagents to compare the results with one performed using A22 Iraq homologous reagents. Therefore, we could not address the issue with one pig #046 that had no detectable antibodies in VNT but was positive throughout the experimental period in the ELISA using A22 Iraq reagents.

## Conclusion

FMD viruses continue to evolve and pose a significant challenge to both endemic and FMD-free countries. Development of new vaccine strains is time consuming and expensive while control of the disease by vaccination using the existing vaccine strains can be a challenge. This study shows that by combining vaccine strains we can increase the efficacy of vaccines against variant FMD viruses. Though these results are based on a small number of pigs and with a virulent virus challenge, we still can get valuable information by employing novel methods of analysis like the AUC method and the Kaplan-Meier probability of survival statistics. Given the epidemiological situation in South-East Asia and the co-circulation of different variants of the A/Asia/SEA-97 lineage, we recommend that both A Malaysia 97 and A22 Iraq 64 are included in the vaccines.

## Data availability statement

The original contributions presented in the study are included in the article/Supplementary material, further inquiries can be directed to the corresponding author.

## Ethics statement

This study was approved by the Australian Centre for Disease Preparedness (ACDP) Animal Ethics Committee (AEC 1774 and AEC 1801), the Canadian Centre for Human and Animal Health Animal Care Committee (AUD# C-15-007), and performed in strict accordance with the recommendations of the Australian Code of Practice for the Care and Use of Animals for Scientific Purposes and the Canadian Council for Animal Care Guidelines.

## Author contributions

WV, NSB, and JH: conceptualization, formal analysis, and writing–original draft preparation. JH and NSB: methodology and data curation. JH and HB: animal studies. NSB, JH, CKN, and WV: writing–review and editing. WV: project administration and funding acquisition. All authors have read and agreed to the published version of the manuscript.

## Funding

This project is supported by Meat and Livestock Australia (MLA; P.PSH.0779), through funding from the Australian Government Department of Agriculture, Fisheries and Forestry as part of its Rural R&D for Profit program (RRND4P-15-02-032), and by producer levies from Australian FMD-susceptible livestock (cattle, sheep, goats, and pigs) industries and Charles Sturt University (CSU), leveraging significant in-kind support from the research partners. The research partners for this project are the Commonwealth Science and Industrial Research Organisation (CSIRO), CSU through the Graham Centre for Agricultural Innovation, the Bureau of Meteorology (BOM), and the Australian Department of Agriculture, Water and the Environment, supported by Animal Health Australia (AHA).

## Conflict of interest

The authors declare that the research was conducted in the absence of any commercial or financial relationships that could be construed as a potential conflict of interest.

## Publisher's note

All claims expressed in this article are solely those of the authors and do not necessarily represent those of their affiliated organizations, or those of the publisher, the editors and the reviewers. Any product that may be evaluated in this article, or claim that may be made by its manufacturer, is not guaranteed or endorsed by the publisher.
