# Supplementary material for: Investigation into the protective ability of monovalent and bivalent A Malaysia 97 and A22 Iraq 64 vaccine strains against infection with an A/Asia/SEA-97 variant in pigs
Source: Front Vet Sci. 2022 Oct 28;9:1027556. doi: 10.3389/fvets.2022.1027556 (PMC9649919; doi:10.3389/fvets.2022.1027556)
Supplement: Supplementary file 1 [file Data_Sheet_1.docx]

**Supplementary Materials:**

**Supplementary Tables**

**Supplementary Table 1: Summary of the groups and their treatments.**

| **Vaccine** | **Group** | **Day of vaccination** | **Challenge** | **No. of pigs** | **Pig IDs** |
| --- | --- | --- | --- | --- | --- |
| A Malaysia 97 | A May V21 | -21 dpc* | Yes | 5 | 001, 002, 003, 004, 005 |
|  | A May V7 | -7 dpc | Yes | 5 | 011, 012, 013, 014, 015 |
|  | A May VO | -21 dpc | No | 2 | 026, 027 |
| A22 Iraq 64 | A22 IRQ V21 | -21 dpc | Yes | 5 | 006, 007, 008, 009, 010 |
|  | A22 IRQ V7 | -7 dpc | Yes | 5 | 016, 017, 018, 019, 020 |
|  | A22 VO | -21 dpc | No | 2 | 028, 029 |
| A Malaysia 97 + A22 Iraq 64 | Combo V21 | -21 dpc | Yes | 5 | 030, 031, 032, 033, 034 |
|  | Combo V7 | -7 dpc | Yes | 5 | 035, 036, 037, 038, 039 |
|  | Combo VO | -21 dpc | No | 5 | 045, 056, 047, 048, 049 |
| Controls | UVC | Unvaccinated | Yes | 10 | 021, 022, 023, 024, 025, 049, 041, 042, 043, 044 |

**-21 dpc – vaccinated 21 days before challenge; -7 dpc – vaccinated 7 days before challenge**

**Supplementary Table 2a: Kaplan-Meier survival plots for protection against A/TAI/15/2013 virus in pigs vaccinated with A May 97 and A22 Iraq 64 vaccines in monovalent or combination formulations. The mean and median protection time post-challenge in days with SE and 95% CI are presented.**

| **Groups** | **records** | **n.max** | **n.start** | **events** | **mean** | **SE** | **median** | **95% CI** |
| --- | --- | --- | --- | --- | --- | --- | --- | --- |
| A May V21 | 75 | 75 | 75 | 46 | 9.88 | 0.40 | 10 | 9-11 |
| A22 V21 | 75 | 75 | 75 | 9 | 13.14 | 0.36 | 14 | 14-∞ |
| Combo V21 | 75 | 75 | 75 | 1 | 14.00 | 0.00 | ∞ | ∞ |
| A May V7 | 75 | 75 | 75 | 51 | 9.40 | 0.41 | 9 | 8-11 |
| A22 V7 | 75 | 75 | 75 | 39 | 10.31 | 0.42 | 11 | 9-12 |
| Combo V7 | 75 | 75 | 75 | 50 | 9.50 | 0.41 | 9.5 | 8-11 |
| UVC | 60 | 60 | 60 | 17 | 5.16 | 0.58 | 5 | 4-5 |

**Supplementary Table 2b: Kaplan-Meier survival plots for protection against A/TAI/15/2013 virus in pigs vaccinated with A May 97 and A22 Iraq 64 vaccines in monovalent or combination formulations. The mean probability of protection (95% CI) on different days post-challenge in vaccinated and unvaccinated pigs are presented.**

| dpc | A May V21 | A22 V21 | Combo V21 | A May V7 | A22 V7 | Combo V7 | UVC |
| --- | --- | --- | --- | --- | --- | --- | --- |
| 3 |  |  |  |  |  |  | 0.97  (0.90-1.00) |
| 4 | 0.98  (0.95-1.00) |  |  | 0.98  (0.95-1.00) |  |  | 0.63  (0.45-0.87) |
| 5 | 0.94  (0.88-1.00) | 0.98  (0.94-1) |  | 0.88  (0.80-0.98) | 0.94  (0.88-1.00) | 0.90  (0.82-0.99) | 0.06  (0.01-0.42) |
| 6 | 0.88  (0.79-0.98) | 0.96  (0.90-1.00) |  | 0.79  (0.68-0.91) | 0.88  (0.79-0.97) | 0.80  (0.70-0.92) |  |
| 7 | 0.77  (0.66-0.90) | 0.89  (0.80-0.99) |  | 0.69  (0.57-0.83) | 0.79  (0.68-0.92) | 0.70  (0.58-0.84) |  |
| 8 | 0.66  (0.54-0.81) |  |  | 0.59  (0.47-0.74) | 0.70  (0.58-0.85) | 0.60  (0.48-0.75) |  |
| 9 | 0.55  (0.42-0.71) |  |  | 0.49  (0.37-0.65) | 0.61  (0.48-0.77) | 0.50  (0.38-0.66) |  |
| 10 | 0.44  (0.32-0.61) |  |  | 0.39  (0.28-0.55) | 0.51  (0.38-0.68) | 0.40  (0.28-0.56) |  |
| 11 | 0.33  (0.22-0.50) |  |  | 0.29  (0.19-0.45) | 0.41  (0.28-0.59) | 0.30  (0.20-0.46) |  |
| 12 | 0.22  (0.13-0.38) |  |  | 0.20  (0.11-0.34) | 0.30  (0.19-0.48) | 0.20  (0.11-0.35) |  |
| 13 | 0.11  (0.05-0.25) |  |  | 0.10  (0.04-0.23) | 0.18  (0.09-0.36) | 0.10  (0.04-0.23) |  |
| 14 | 0  (NA-NA) | 0.18  (0.03-1) | 0.8  (0.52-1.00) | 0  (NA-NA) | 0  (NA-NA) | 0  (NA-NA) |  |

**Supplementary Table 3a: Neutralising antibody response in pigs against A May 97 vaccine strain. Titres less than 1.20 log_10_ (1:16) were considered negative. Bold indicates a positive tire. Cross neutralising antibodies for A22 vaccine groups were not tested against A May 97 vaccine strain.**

| **Vaccine group** | **Pig ID** | **-21/-7 dpc^†^** | **0 dpc** | **5 dpc** | **7 dpc** | **10 dpc** | **14 dpc** |
| --- | --- | --- | --- | --- | --- | --- | --- |
| **A May 21** | 001 | <0.90 | <0.90 | E | E | E | E |
|  | 002 | <0.90 | <0.90 | **1.51** | E | E | E |
|  | 003 | <0.90 | <0.90 | **1.38** | **1.68** | E | E |
|  | 004 | <0.90 | <0.90 | <0.90 | 0.90 | E | E |
|  | 005 | <0.90 | <0.90 | <0.90 | E | E | E |
| **Combo V21** | 030 | <0.90 | <0.90 | **1.20** | **1.81** | **1.68** | **1.98** |
|  | 031 | <0.90 | <0.90 | **1.20** | **1.38** | **1.98** | **2.11** |
|  | 032 | <0.90 | <0.90 | **1.81** | **2.71** | **2.71** | **3.0** |
|  | 033 | <0.90 | <0.90 | 0.90 | **1.98** | **2.11** | **2.28** |
|  | 034 | <0.90 | <0.90 | 1.08 | **1.98** | **2.41** | **1.98** |
| **A May V7** | 011 | <0.90 | <0.90 | 0.90 | E | E | E |
|  | 012 | <0.90 | <0.90 | 0.90 | E | E | E |
|  | 013 | <0.90 | <0.90 | <0.90 | E | E | E |
|  | 014 | <0.90 | <0.90 | <0.90 | E | E | E |
|  | 015 | <0.90 | <0.90 | E | E | E | E |
| **Combo V7** | 035 | <0.90 |  | <0.90 | E | E | E |
|  | 036 | <0.90 |  | <0.90 | E | E | E |
|  | 037 | <0.90 |  | <0.90 | E | E | E |
|  | 038 | <0.90 |  | <0.90 | E | E | E |
|  | 039 | <0.90 |  | <0.90 | E | E | E |
| **UVC** | 021 |  | <0.90 | E | E | E | E |
|  | 022 |  | <0.90 | E | E | E | E |
|  | 023 |  | <0.90 | E | E | E | E |
|  | 024 |  | <0.90 | E | E | E | E |
|  | 025 |  | <0.90 | E | E | E | E |
|  | 040 |  | <0.90 | E | E | E | E |
|  | 041 |  | <0.90 | E | E | E | E |
|  | 042 |  | <0.90 | <0.90 | E | E | E |
|  | 043 |  | <0.90 | <0.90 | E | E | E |
|  | 044 |  | <0.90 | E | E | E | E |
| **A May VO** | 026 | <0.90 |  | <0.90 | <0.90 | <0.90 | <0.90 |
|  | 027 | <0.90 |  | <0.90 | <0.90 | <0.90 | 0.90 |
| **Combo VO** | 045 | <0.90 | <0.90 | <0.90 | <0.90 | **1.38** | 1.08 |
|  | 046 | <0.90 | <0.90 | <0.90 | <0.90 | <0.90 | 0.90 |
|  | 047 | <0.90 | <0.90 | <0.90 | 0.90 | 1.08 | **1.51** |
|  | 048 | <0.90 | 1.08 | **1.38** | **1.51** | **1.68** | **1.98** |
|  | 049 | <0.90 | <0.90 | <0.90 | **1.20** | **1.38** | **1.20** |

**^†^**dpc = day post-challenge; E = Euthanised

**Supplementary Table 3b: Neutralising antibody response in pigs against A22 Iraq 64 vaccine strain. Titres less than 1.20 log_10_ (1:16) were considered negative. Bold indicates a positive tire. Cross neutralising antibodies for A May vaccine groups were not tested against A22 Iraq 64 vaccine strain.**

| **Vaccine group** | **Pig ID** | **-21/-7 dpc^†^** | **0 dpc** | **5 dpc** | **7 dpc** | **10 dpc** | **14 dpc** |
| --- | --- | --- | --- | --- | --- | --- | --- |
| **A22 V21** | 006 | <0.90 | <0.90 | **1.68** | **1.68** | E | E |
|  | 007 | <0.90 | <0.90 | 0.90 | **1.20** | **1.68** | **1.68** |
|  | 008 | <0.90 | <0.90 | **1.51** | **1.98** | **1.51** | **1.98** |
|  | 009 | <0.90 | <0.90 | <0.90 | E | E | E |
|  | 010 | <0.90 | <0.90 | **1.68** | **1.68** | E | E |
| **Combo V21** | 030 | <0.90 | <0.90 | **1.38** | **1.81** | **2.11** | **1.81** |
|  | 031 | <0.90 | <0.90 | <0.90 | 1.08 | **1.51** | **1.81** |
|  | 032 | <0.90 | <0.90 | **1.20** | **1.68** | **1.98** | **2.28** |
|  | 033 | <0.90 | <0.90 | <0.90 | **1.20** | **1.38** | **1.68** |
|  | 034 | <0.90 | <0.90 | 0.90 | **1.38** | **1.51** | **1.81** |
| **A22 V7** | 016 | <0.90 | <0.90 | <0.90 | E | E | E |
|  | 017 | <0.90 | <0.90 | 0.90 | E | E | E |
|  | 018 | <0.90 | <0.90 | <0.90 | **1.68** | **1.20** | **1.68** |
|  | 019 | <0.90 | <0.90 | <0.90 | 1.08 | E | E |
|  | 020 | <0.90 | <0.90 | **1.20** | E | E | E |
| **Combo V7** | 035 | <0.90 |  | <0.90 | E | E | E |
|  | 036 | <0.90 |  | <0.90 | E | E | E |
|  | 037 | <0.90 |  | <0.90 | E | E | E |
|  | 038 | <0.90 |  | <0.90 | E | E | E |
|  | 039 | <0.90 |  | <0.90 | E | E | E |
| **UVC** | 021 |  | <0.90 | E | E | E | E |
|  | 022 |  | <0.90 | E | E | E | E |
|  | 023 |  | <0.90 | E | E | E | E |
|  | 024 |  | <0.90 | E | E | E | E |
|  | 025 |  | <0.90 | E | E | E | E |
|  | 040 |  | <0.90 | E | E | E | E |
|  | 041 |  | <0.90 | E | E | E | E |
|  | 042 |  | <0.90 | <0.90 | E | E | E |
|  | 043 |  | <0.90 | <0.90 | E | E | E |
|  | 044 |  | <0.90 | E | E | E | E |
| **A22 VO** | 028 | <0.90 |  | <0.90 | 0.90 | 1.08 | 0.90 |
|  | 029 | <0.90 |  | <0.90 | **1.20** | 0.9 | **1.68** |
| **Combo VO** | 045 | <0.90 | <0.90 | <0.90 | <0.90 | <0.90 | <0.90 |
|  | 046 | <0.90 | <0.90 | <0.90 | 0.90 | <0.90 | 1.08 |
|  | 047 | <0.90 | <0.90 | <0.90 | 0.90 | <0.90 | **1.20** |
|  | 048 | <0.90 | <0.90 | 0.90 | **1.20** | **1.38** | **1.68** |
|  | 049 | <0.90 | <0.90 | <0.90 | <0.90 | 0.90 | **1.51** |

**^†^**dpc = day post-challenge; E = Euthanised

**Supplementary Table 3c: Neutralising antibody response in pigs against A TAI/15/2013 challenge strain. Titres less than 1.20 log_10_ (1:16) were considered negative. Bold indicates a positive tire.**

| **Vaccine group** | **Pig ID** | **-21/-7 dpc^†^** | **0 dpc** | **5 dpc** | **7 dpc** | **10 dpc** | **14 dpc** |
| --- | --- | --- | --- | --- | --- | --- | --- |
| **A May V21** | 001 | <0.90 | <0.90 | E | E | E | E |
|  | 002 | <0.90 | <0.90 | <0.90 | E | E | E |
|  | 003 | <0.90 | <0.90 | 0.90 | **1.68** | E | E |
|  | 004 | <0.90 | <0.90 | 0.90 | **1.20** | E | E |
|  | 005 | <0.90 | <0.90 | 1.08 | E | E | E |
| **A22 V21** | 006 | <0.90 | <0.90 | **1.51** | **1.98** | E | E |
|  | 007 | <0.90 | <0.90 | <0.90 | **1.51** | **1.68** | **1.68** |
|  | 008 | <0.90 | <0.90 | **1.51** | **2.41** | **2.41** | **2.89** |
|  | 009 | <0.90 | <0.90 | 0.90 | E | E | E |
|  | 010 | <0.90 | <0.90 | 1.08 | **2.11** | E | E |
| **Combo V21** | 030 | <0.90 | <0.90 | **1.38** | **1.51** | **1.81** | **1.81** |
|  | 031 | <0.90 | <0.90 | 0.90 | **1.68** | **1.81** | **1.98** |
|  | 032 | <0.90 | <0.90 | **1.68** | **2.71** | **2.58** | **2.41** |
|  | 033 | <0.90 | <0.90 | **1.20** | **1.68** | **1.81** | **1.98** |
|  | 034 | <0.90 | <0.90 | **1.20** | **1.81** | **2.11** | **2.11** |
| **A May V7** | 011 | <0.90 | <0.90 | 0.90 | E | E | E |
|  | 012 | <0.90 | <0.90 | <0.90 | E | E | E |
|  | 013 | <0.90 | <0.90 | 1.08 | E | E | E |
|  | 014 | <0.90 | <0.90 | <0.90 | E | E | E |
|  | 015 | <0.90 | <0.90 | E | E | E | E |
| **A22 V7** | 016 | <0.90 | <0.90 | <0.90 | E | E | E |
|  | 017 | <0.90 | <0.90 | <0.90 | E | E | E |
|  | 018 | <0.90 | <0.90 | <0.90 | 0.90 | **1.51** | **1.68** |
|  | 019 | <0.90 | <0.90 | <0.90 | **1.20** | E | E |
|  | 020 | <0.90 | <0.90 | <0.90 | E | E | E |
| **Combo V7** | 035 | <0.90 | <0.90 | <0.90 | E | E | E |
|  | 036 | <0.90 | <0.90 | <0.90 | E | E | E |
|  | 037 | <0.90 | <0.90 | <0.90 | E | E | E |
|  | 038 | <0.90 | <0.90 | <0.90 | E | E | E |
|  | 039 | <0.90 | <0.90 | <0.90 | E | E | E |
| **UVC** | 021 |  | <0.90 | E | E | E | E |
|  | 022 |  | <0.90 | E | E | E | E |
|  | 023 |  | <0.90 | E | E | E | E |
|  | 024 |  | <0.90 | E | E | E | E |
|  | 025 |  | <0.90 | E | E | E | E |
|  | 040 |  | <0.90 | E | E | E | E |
|  | 041 |  | <0.90 | E | E | E | E |
|  | 042 |  | <0.90 | <0.90 | E | E | E |
|  | 043 |  | <0.90 | <0.90 | E | E | E |
|  | 044 |  | <0.90 | E | E | E | E |
| **A May VO** | 026 | <0.90 |  | <0.90 | <0.90 | <0.90 | <0.90 |
|  | 027 | <0.90 |  | <0.90 | <0.90 | <0.90 | <0.90 |
| **A22 VO** | 028 | <0.90 |  | <0.90 | 0.90 | **1.20** | 0.90 |
|  | 029 | <0.90 |  | <0.90 | <0.90 | <0.90 | **1.81** |
| **Combo VO** | 045 | <0.90 | <0.90 | <0.90 | <0.90 | **1.38** | 1.08 |
|  | 046 | <0.90 | <0.90 | <0.90 | <0.90 | <0.90 | 1.08 |
|  | 047 | <0.90 | <0.90 | <0.90 | 0.90 | <0.90 | **1.38** |
|  | 048 | <0.90 | <0.90 | 0.90 | **1.20** | 1.08 | **1.20** |
|  | 049 | <0.90 | <0.90 | <0.90 | <0.90 | <0.90 | 1.08 |

**^†^**dpc = day post-challenge; E = Euthanised

**Supplementary Table 4: Antibodies to FMDV serotype A (A22) structural proteins by solid-phase competition ELISA (SPCE). Percent inhibition values are shown for different days post-challenge^†^; % inhibition >50 is considered positive (indicated in grey). E = animal had been euthanized.**

| **Group** | **Pig ID** | **-21/-7 dpc^†^** | **0 dpc** | **4 dpc** | **5 dpc** | **6 dpc** | **7 dpc** | **14 dpc** |
| --- | --- | --- | --- | --- | --- | --- | --- | --- |
| A May V21 | 001 | 2.05 | 42.43 | 71.21 | E | E | E | E |
|  | 002 | 2.36 | 55.93 | 71.92 | 85.42 | E | E | E |
|  | 003 | 4.53 | 71.62 | 86.43 | 89.20 | 90.50 | 91.07 | E |
|  | 004 | 1.87 | 15.49 | 62.73 | 78.07 | 86.07 | 88.50 | E |
|  | 005 | 8.23 | 44.30 | 67.58 | 82.30 | 90.21 | E | E |
| A22 V21 | 006 | 7.91 | 26.19 | 66.36 | 82.15 | 87.63 | 89.19 | E |
|  | 007 | 3.86 | 22.56 | 50.73 | 71.12 | 82.50 | 87.75 | E |
|  | 008 | 3.43 | 30.31 | 60.36 | 80.01 | 87.48 | 89.80 | E |
|  | 009 | 8.67 | 33.56 | 55.01 | 66.79 | E | E | E |
|  | 010 | 4.48 | 26.60 | 58.03 | 79.21 | 85.62 | 88.97 | E |
| Combo V21 | 030 | -42.77 | 56.17 | 70.78 | 75.11 | 81.23 | 80.46 | 75.01 |
|  | 031 | -36.76 | 59.41 | 77.38 | 55.85 | 86.68 | 74.55 | 52.13 |
|  | 032 | -54.75 | 80.62 | 81.95 | 86.81 | 85.23 | 72.55 | 65.25 |
|  | 033 | -14.13 | 66.89 | 68.54 | 81.83 | 84.23 | 74.97 | 85.64 |
|  | 034 | 2.17 | 77.38 | 76.39 | 84.45 | 77.00 | 79.78 | 85.06 |
| V7 May | 011 | 13.72 | 29.55 | 69.75 | 78.92 | E | E | E |
|  | 012 | 15.56 | 30.86 | 72.59 | 79.03 | E | E | E |
|  | 013 | 1.61 | 29.87 | 71.85 | 80.89 | E | E | E |
|  | 014 | -1.25 | 34.68 | 72.15 | 81.60 | E | E | E |
|  | 015 | 2.37 | 25.13 | 53.33 | E | E | E | E |
| A22 V7 | 016 | 2.07 | 19.82 | 61.59 | 78.00 | E | E | E |
|  | 017 | 0.15 | 19.11 | 61.59 | 75.10 | E | E | E |
|  | 018 | 0.68 | 21.10 | 32.45 | 57.72 | 71.87 | 75.94 | 55.27 |
|  | 019 | 2.33 | 23.74 | 38.75 | 64.99 | 77.23 | 78.90 | E |
|  | 020 | 1.21 | 7.23 | 65.36 | 78.13 | E | E | E |
| Combo V7 | 035 | 0.02 | 42.93 | 50.12 | 66.09 | E | E | E |
|  | 036 | 3.97 | 41.60 | 37.32 | 48.66 | E | E | E |
|  | 037 | 0.05 | 39.80 | 42.39 | 49.72 | E | E | E |
|  | 038 | 1.43 | 48.74 | 53.60 | 70.89 | E | E | E |
|  | 039 | 3.38 | 42.88 | 58.20 | 55.34 | E | E | E |
| UV | 021 | ND | -0.60 | 32.84 | E | E | E | E |
|  | 022 | ND | -1.76 | 42.41 | E | E | E | E |
|  | 023 | ND | -0.61 | 56.69 | E | E | E | E |
|  | 024 | ND | 2.16 | 37.20 | E | E | E | E |
|  | 025 | ND | 6.14 | 47.30 | E | E | E | E |
|  | 040 | ND | -17.66 | 8.84 | E | E | E | E |
|  | 041 | ND | 6.46 | 49.41 | E | E | E | E |
|  | 042 | ND | 18.45 | 45.98 | 67.93 | E | E | E |
|  | 043 | ND | 0.59 | 76.30 | 72.27 | E | E | E |
|  | 044 | ND | 11.17 | 43.26 |  | E | E | E |
| A May VO | 026 | 1.44 | 36.72 | 47.24 | 47.13 | 52.91 | 50.88 | 56.04 |
|  | 027 | 5.97 | 56.28 | 69.81 | 66.32 | 74.47 | 75.45 | 81.49 |
| A22 VO | 028 | 5.99 | 66.54 | 64.94 | 65.71 | 69.53 | 72.78 | 81.55 |
|  | 029 | 3.95 | 21.42 | 24.48 | 31.14 | 27.29 | 28.55 | 35.24 |
| Combo VO | 045 | 3.69 | 25.97 | 35.42 | 36.08 | 19.77 | 44.18 | 60.26 |
|  | 046 | 1.25 | 54.30 | 64.31 | 57.57 | 60.39 | 54.72 | 71.76 |
|  | 047 | -3.21 | 64.56 | 56.12 | 53.86 | 50.21 | 61.69 | 68.73 |
|  | 048 | -6.96 | 73.59 | 42.13 | 76.56 | 68.75 | 79.48 | 83.29 |
|  | 049 | -7.18 | 72.53 | 58.92 | 36.09 | 70.73 | 72.07 | 77.58 |

**Supplementary Table 5a: Median and Mean AUC values for pigs determined based on the RNA levels in blood swabs**

| Pigs | Median AUC values | | | | | | | Median AUC values | | | | | | |
| --- | --- | --- | --- | --- | --- | --- | --- | --- | --- | --- | --- | --- | --- | --- |
|  | A May V21 | A22 V21 | Combo V21 | A May V7 | A22 V7 | Combo V7 | UVC | A May V21 | A22 V21 | Combo V21 | A May V7 | A22 V7 | Combo V7 | UVC |
| P1 | 126.12 | 0.00 | 66.60 | 0.00 | 0.00 | 0.00 | 121.68 | 107.71 | 18.05 | 76.03 | 18.05 | 21.65 | 0.00 | 105.17 |
| P2 | 94.56 | 0.00 | 45.12 | 0.00 | 0.00 | 55.20 | 146.04 | 83.33 | 18.14 | 36.38 | 0.00 | 0.00 | 45.41 | 128.02 |
| P3 | 0.00 | 0.00 | 53.88 | 0.00 | 0.00 | 55.80 | 127.08 | 18.05 | 0.00 | 48.05 | 19.63 | 0.00 | 45.89 | 120.29 |
| P4 | 67.80 | 68.28 | 0.00 | 0.00 | 0.00 | 0.00 | 130.80 | 84.19 | 73.44 | 0.00 | 18.29 | 0.00 | 21.07 | 116.30 |
| P5 | 0.00 | 0.00 | 67.68 | 55.92 | 0.00 | 61.08 | 73.56 | 0.00 | 18.05 | 56.69 | 48.77 | 0.00 | 73.58 | 96.82 |
| P6 |  |  |  |  |  |  | 86.16 |  |  |  |  |  |  | 106.03 |
| P7 |  |  |  |  |  |  | 136.56 |  |  |  |  |  |  | 132.67 |
| P8 |  |  |  |  |  |  | 157.80 |  |  |  |  |  |  | 147.41 |
| P9 |  |  |  |  |  |  | 112.44 |  |  |  |  |  |  | 129.31 |
| P10 |  |  |  |  |  |  | 114.12 |  |  |  |  |  |  | 117.02 |

**Supplementary Table 5b: Median and Mean AUC values for pigs determined based on the RNA levels in nasal swabs**

| Pigs | Median AUC values | | | | | | | Median AUC values | | | | | | |
| --- | --- | --- | --- | --- | --- | --- | --- | --- | --- | --- | --- | --- | --- | --- |
|  | A May V21 | A22 V21 | Combo V21 | A May V7 | A22 V7 | Combo V7 | UVC | A May V21 | A22 V21 | Combo V21 | A May V7 | A22 V7 | Combo V7 | UVC |
| P1 | 56.70 | 54.13 | 67.22 | 56.39 | 0.00 | 63.49 | 0.00 | 66.43 | 68.00 | 83.04 | 72.20 | 26.99 | 58.44 | 0.00 |
| P2 | 70.86 | 59.40 | 0.00 | 58.55 | 0.00 | 67.70 | 45.82 | 82.94 | 68.92 | 49.86 | 56.52 | 28.14 | 61.45 | 60.65 |
| P3 | 50.45 | 53.23 | 66.24 | 61.27 | 0.00 | 76.15 | 0.00 | 69.18 | 64.31 | 71.17 | 57.92 | 27.48 | 76.74 | 55.03 |
| P4 | 123.98 | 73.58 | 47.82 | 51.31 | 56.31 | 81.93 | 47.24 | 95.23 | 79.42 | 68.34 | 58.76 | 54.54 | 87.18 | 66.66 |
| P5 | 111.71 | 55.28 | 0.00 | 60.79 | 0.00 | 56.16 | 0.00 | 104.25 | 75.25 | 51.92 | 71.87 | 32.30 | 63.67 | 54.86 |
| P6 |  |  |  |  |  |  | 0.00 |  |  |  |  |  |  | 49.75 |
| P7 |  |  |  |  |  |  | 62.10 |  |  |  |  |  |  | 63.46 |
| P8 |  |  |  |  |  |  | 70.15 |  |  |  |  |  |  | 68.68 |
| P9 |  |  |  |  |  |  | 70.71 |  |  |  |  |  |  | 69.80 |
| P10 |  |  |  |  |  |  | 0.00 |  |  |  |  |  |  | 52.07 |

**Supplementary Table 5c: Median and Mean AUC values for pigs determined based on the RNA levels in oral swabs**

| Pigs | Median AUC values | | | | | | | Median AUC values | | | | | | |
| --- | --- | --- | --- | --- | --- | --- | --- | --- | --- | --- | --- | --- | --- | --- |
|  | A May V21 | A22 V21 | Combo V21 | A May V7 | A22 V7 | Combo V7 | UVC | A May V21 | A22 V21 | Combo V21 | A May V7 | A22 V7 | Combo V7 | UVC |
| P1 | 50.40 | 54.31 | 62.63 | 0.00 | 52.23 | 68.34 | 0.00 | 67.21 | 68.96 | 72.65 | 45.02 | 64.03 | 59.51 | 54.47 |
| P2 | 67.76 | 103.61 | 77.96 | 67.64 | 55.63 | 68.81 | 0.00 | 78.05 | 86.12 | 72.68 | 78.43 | 71.02 | 70.19 | 52.25 |
| P3 | 70.72 | 51.07 | 70.38 | 125.38 | 61.20 | 70.94 | 58.54 | 74.63 | 71.03 | 79.03 | 117.46 | 65.19 | 77.26 | 66.25 |
| P4 | 135.54 | 70.40 | 66.95 | 115.69 | 56.65 | 55.85 | 0.00 | 106.42 | 77.07 | 72.02 | 91.26 | 60.45 | 67.43 | 58.57 |
| P5 | 117.35 | 69.72 | 0.00 | 60.34 | 54.98 | 65.59 | 0.00 | 93.94 | 76.03 | 48.44 | 63.85 | 56.43 | 77.30 | 51.77 |
| P6 |  |  |  |  |  |  | 0.00 |  |  |  |  |  |  | 50.67 |
| P7 |  |  |  |  |  |  | 62.62 |  |  |  |  |  |  | 67.13 |
| P8 |  |  |  |  |  |  | 71.36 |  |  |  |  |  |  | 79.59 |
| P9 |  |  |  |  |  |  | 57.28 |  |  |  |  |  |  | 63.01 |
| P10 |  |  |  |  |  |  | 69.97 |  |  |  |  |  |  | 69.12 |

**Supplementary Table 6a: Summary of one-way ANOVA with all groups including unvaccinated animals included**

| **AUC** | **Sample** | **F value** | **Pr(>F)** | **Signif.^†^** | **Post hoc Bonferroni adjusted p-value** |
| --- | --- | --- | --- | --- | --- |
| Median | Viraemia | 13.41 | 1.20E-07 | *** | A22 V21 vs UVC: <0.0001; A22 V7 vs UVC: <0.0001; Combo V21 vs UVC: <0.0001; Combo V7 - UVC: <0.001; A May V21 - UVC: <0.05; A May V7 - UVC: <0.01 |
|  | Oral Swabs | 2.37 | 0.0517 | . | A May V21 vs UVC: <0.05 |
|  | Nasal Swabs | 5.106 | 0.000835 | *** | Combo V21 vs A May V21: <0.05; Combo V21 vs A May V21: <0.01; A May V21 vs UVC: p<0.05 |
| Mean | Viraemia | 16.98 | 7.82E-09 | *** | A22 V21 vs UVC: <0.0001; A22 V7 vs UVC: <0.0001; Combo V21 vs UVC: <0.0001; Combo V7 - UVC: <0.0001; A May V21 - UVC: <0.01; A May V7 - UVC: <0.001 |
|  | Oral Swabs | 2.38 | 0.0508 | . | A May V21 - UVC: <0.10 |
|  | Nasal Swabs | 5.961 | 0.000267 | *** | Combo V21 vs A22 V21: <0.01; Combo V21 vs A22 V7: <0.05; Combo V21 vs Combo 7: p<0.01; Combo V21 vs A May V21: <0.001; A May V21 vs UVC: <0.05 |

^†^Significance codes for p-values: ‘***’ <0.001; ‘**’ <0.01; ‘*’ <0.05; ‘.’ <0.1

**Supplementary Table 6b: Summary of two-way ANOVA without unvaccinated animals**

| **AUC** | **Sample** | **F value** | **Pr(>F)^†^** | **Signif. ^†^** | **Post hoc Tukey's HSD adjusted p-values** |
| --- | --- | --- | --- | --- | --- |
| Median | Viraemia | 2.338 | 0.0728^α^ | . | Combo V21 vs A May V21: p<0.10 |
|  | Oral Swabs | 0.848 | 0.529 | NS |  |
|  | Nasal Swabs | 6.279 | 0.000738^β^ | *** | Combo V21 vs A22 V21: p<0.05; Combo V21 vs A22 V7: p<0.05; Combo V7 vs Combo V21: p<0.01; May97 V21 vs Combo V21: p<0.001; May97 V7 vs May97 V21: p<0.05 |
| Mean | Viraemia | 2.18 | 0.0901 | . | Combo V21 vs A May V21: p<0.10 |
|  | Oral Swabs | 1.324 | 0.288 | NS |  |
|  | Nasal Swabs | 9.773 | 3.43E-05 | *** | Combo V21 vs A22 V21: p<0.001; Combo V21 vs A22 V7: p<0.01; Combo V7 vs Combo V21: p<0.01; A May V21 vs Combo V21: p<0.001; A May V7 vs Combo V21: p<0.10 |
